# Zeaxanthin and Lutein in the Management of Eye Diseases

**DOI:** 10.1155/2016/4915916

**Published:** 2016-04-07

**Authors:** Shun-Fa Yang, Joan E. Roberts, Qing-huai Liu, Jijing Pang, Tadeusz Sarna

**Affiliations:** ^1^Institute of Medicine, Chung Shan Medical University, Taichung 40256, Taiwan; ^2^Department of Medical Research, Chung Shan Medical University Hospital, Taichung 40256, Taiwan; ^3^Department of Natural Sciences, Fordham University, New York, NY 10458, USA; ^4^Department of Ophthalmology, The First Affiliated Hospital of Nanjing Medical University, Nanjing 210029, China; ^5^Department of Ophthalmology, College of Medicine, University of Florida, FL 32610, USA; ^6^Department of Biophysics, Faculty of Biochemistry, Biophysics, and Biotechnology, Jagiellonian University in Krakow, 31-007 Krakow, Poland

Zeaxanthin and lutein, two carotenoid pigments of the xanthophyll subclass, are present in a high concentration in the retina, especially in the macula. They work as a filter protecting the macula from the blue light and also as structurally bound antioxidants which protect surrounding ocular cells against oxidative stress. Many observational and interventional studies have indicated that lutein and zeaxanthin might reduce the risk of various eye diseases, especially the age-related macular degeneration.

In this special issue, four review articles discuss zeaxanthin and lutein in the context of basic science studies, experimental animal studies, clinical trials, and safety and toxicological studies. J. E. Roberts and J. Dennison, in their paper “The Photobiology of Lutein and Zeaxanthin in the Eye,” reviewed the oxidative stress inherently in photobiology, the reactive intermediate(s) of endogenous or exogenous photosensitizing agents in ocular tissues, and the ability of zeaxanthin and lutein to protect ocular tissues against damage. C. Xue et al. published the paper “Management of Ocular Diseases Using Lutein and Zeaxanthin: What Have We Learned from Experimental Animal Studies?” which reviews the preventive and therapeutic effects of zeaxanthin and lutein on various ocular diseases as studied in experimental animal models. This comprehensive survey provides new insights on future use of these xanthophylls for clinical management of vision-threatening diseases. N. K. Scripsema et al. authored a review article, “Lutein, Zeaxanthin, and* meso*-Zeaxanthin in the Clinical Management of Eye Disease,” which looks at the current collection of epidemiological studies and clinical trials of carotenoids in various ocular diseases. These studies, especially the powerful randomized, placebo-controlled clinical trials, have confirmed the ability of zeaxanthin and lutein to modify the visual loss and risk of progression to advanced AMD, especially neovascular AMD. J. A. Edwards published an article, “Zeaxanthin: Review of Toxicological Data and Acceptable Daily Intake,” which further explores zeaxanthin's safety in numerous well-organized experimental animal studies, which support its clinical use at much higher doses than what are currently recommended for the management of AMD.

Recent studies have revealed that, in addition to traditional mechanisms, lutein and zeaxanthin can influence the viability and function of cells through various signal pathways or transcription factors, such as their ability to inhibit the growth and cause apoptosis of malignant tumor cells such as ocular melanoma cells. In this issue, X. L. Xu, from the Memorial Sloan-Kettering Cancer Center, collaborated with researchers and pathologists from New York Eye and Ear infirmary to explore the “Effects of Zeaxanthin on Growth and Invasion of Human Uveal Melanoma in Nude Mouse Model.” The study documents the ability of intraocularly administered zeaxanthin to significantly inhibit the growth and invasion of human uveal melanoma in nude mice. M.-C. Bi et al. in their paper, “Nonlethal Levels of Zeaxanthin Inhibit Cell Migration, Invasion, and Secretion of MMP-2 via NF-*κ*B Pathway in Cultured Human Uveal Melanoma Cells,” reported that zeaxanthin inhibited the secretion of MMP-2 along with the migration and invasion of cultured human uveal melanoma cells. Both melanoma papers suggest that zeaxanthin may be a promising agent in the management of uveal melanoma and prevention of its spread.

The role of lutein and zeaxanthin in management of inflammation is explored by S.-C. Chao et al. and H.-Y. Lin et al. in their two papers: “Effects of Lutein and Zeaxanthin on LPS-induced Secretion of IL-8 by Uveal Melanocytes and Relevant Signal Pathways” and “Effects of Lutein on Hyperosmoticity-Induced Upregulation of IL-6 in Cultured Corneal Epithelial Cells and Its Relevant Signal Pathways.” The first paper describes the ability of zeaxanthin and lutein to inhibit LPS-induced secretion of IL-8 by uveal melanocytes, which suggests a potential role for their application in the management of uveitis and other inflammatory eye diseases. The second paper shows how lutein inhibits hyperosmoticity-induced upregulation of IL-6 in cultured corneal epithelial cells and suggests that lutein may be a promising agent for the local treatment of dry eye.

Y. Tian et al. in their paper, “Lutein Leads to a Decrease of Factor D Secretion by Cultured Mature Human Adipocytes,” reported that secretion of Factor D (the rate limiting enzyme of the complement alternative implicated in the pathogenesis of AMD) was significantly decreased following lutein supplementation to cultured human adipocytes. This suggests that lutein could be a useful tool for blocking the progression of AMD and other inflammatory diseases that are modulated by FD.

The one clinical study in this issue by S. M. van der Made et al., “Increased Macular Pigment Optical Density and Visual Acuity following Consumption of a Buttermilk Drink Containing Lutein-Enriched Egg Yolks: A Randomized, Bouble-Blind, Placebo-Controlled Trial,” demonstrated improved visual acuity, macular pigment, and plasma lutein concentrations in elderly subjects with drusen and/or retinal pigment epithelial abnormalities following daily consumption of a dairy drink containing lutein-enriched egg yolks for one year.

Papers in this special issue have been assembled to go beyond the well-documented therapeutic effects of lutein and zeaxanthin on the AMD and explore future applications, suggested by animal and in vitro data, for diabetic retinopathy, cataract, uveal melanoma, phototoxicity, retinal detachment, uveitis, and dry eye. In addition, they demonstrate that the routes of administration of zeaxanthin and lutein could be expanded from oral administration to include local applications, such as eye drops or intravitreal injection. Finally, they point out that the dosages of zeaxanthin and lutein used in clinical trials could be increased dozenfold those currently used, if necessary for greater efficacy in severe conditions such as uveitis or uveal melanoma. It is the hope of the editors and authors that these insights may stimulate new uses for these ubiquitous but largely underappreciated components of our ocular environment.

*Shun-Fa Yang* *Shun-Fa Yang* *Joan E. Roberts* *Joan E. Roberts* *Qing-huai Liu* *Qing-huai Liu* *Jijing Pang* *Jijing Pang* *Tadeusz Sarna* *Tadeusz Sarna*

